# Local problems need global solutions: The metabolic needs of regenerating organisms

**DOI:** 10.1111/wrr.13029

**Published:** 2022-05-21

**Authors:** Ines C. Kübler, Jenny Kretzschmar, Marko Brankatschk, Tatiana Sandoval-Guzmán

**Affiliations:** 1 Center for Regenerative Therapies Dresden, Technische Universität Dresden, Dresden, Germany; 2 MRC Laboratory of Molecular Biology, Cambridge Biomedical Campus, Cambridge, UK; 3 Department of Molecular, Cell and Developmental Biology, Technische Universität Dresden, Dresden, Germany; 4 Department of Internal Medicine III, Center for Healthy Aging, University Hospital Carl Gustav Carus, Technische Universität Dresden, Dresden, Germany; 5 Paul Langerhans Institute Dresden of Helmholtz Centre Munich, University Clinic Carl Gustav Carus, Technische Universität Dresden, Dresden, Germany

**Keywords:** insulin, lipids, metabolism, regeneration, systemic response

## Abstract

The vast majority of species that belong to the plant or animal kingdom evolved with two main strategies to counter tissue damage—scar formation and regeneration. Whereas scar formation provides a fast and cost-effective repair to exit life-threatening conditions, complete tissue regeneration is time-consuming and requires vast resources to reinstall functionality of affected organs or structures. Local environments in wound healing are widely studied and findings have provided important biomedical applications. Less well understood are organismic physiological parameters and signalling circuits essential to maintain effective tissue repair. Here, we review accumulated evidence that positions the interplay of local and systemic changes in metabolism as essential variables modulating the injury response. We particularly emphasise the role of lipids and lipid-like molecules as significant components long overlooked.

## 1 Introduction

The functional restoration of damaged or lost tissues by trauma or disease is an integral component of life and widespread across species. Some animals, like salamanders, possess the unique ability to regenerate an entire limb in a few weeks. Other species like planarians and cnidarians even regenerate an entire body. In contrast, most vertebrates evolved to rapidly repair damages that expose the organism to external hazards. Such repair may result in non-functional tissue replacements and the formation of a fibrous matrix called a scar.^1^

Regeneration in mammals is observed vastly in tissues with high proliferative potential and resident stem cell populations—the liver, blood or intestinal epithelium. Furthermore, decline or depletion of the resident stem cell pool (e.g., due to aging), affects cellular turnover and the regenerative potential.^2^

While the extent of regeneration and the timing differs from organ to species, there is a clear need for (1) new building blocks: amino acids, sugars, lipids and nucleic acids, (2) signals to orchestrate the process and (3) energetic fuel to accomplish it. Despite this, little is known on how a regenerating organism regulates its metabolism. Furthermore, and often overlooked, regeneration should not be seen as an isolated process, but rather influenced by the circumstances of the organism at a particular time and place. For example, a salamander that spends at least 6-9 weeks re-making a whole limb, should rebalance its metabolic homeostasis to adapt to this long-term energy demand. However, it remains a mystery how a local injury shifts the energy resources and how the organism modulates it.

Animal and vegetal species regenerate through different mechanisms (reviewed elsewhere,^3–7^) and this evolutionary diversity underlines regeneration as a basic biological property in living organisms. One common initial mechanism is re-epithelization of the wound, to seal the organisms from the external environment. Some organisms go further and form a specialised wound epidermis (WE).^8^ The WE is a multi-layered epithelium, functioning as an important signalling centre during subsequent steps like the formation of a temporal structure called the blastema. The blastema is a highly proliferative cell mass, containing the dedifferentiated cells, as well as recruited resident stem cells which can give rise to all cell types needed for regeneration of the missing body part. Planarians, teleost fish, salamanders, reptiles and mammals among others, regenerate lost body parts through a blastema. In models where the blastema is absent, persistent active migration and proliferation are crucial for the re-formation of the missing structure. However, the requirement of energy to regenerate tissues and complex structures is a common ground.

Although the regulation of blastema formation and pattern configuration is well studied, less is known about how other variables including environment and internal milieu affect the rate and quality of regeneration.^9^ Seasonal climate and food availability are important examples of external cues with a defining influence on the physiology of an organism. While internally, an organism balances energy to contend with age, reproduction status, nutrition and stress.^10^

In this review, we emphasise the—even less studied—interplay of regeneration and the recruitment of energy resources during a sustained repair. To use energy efficiently, an organism in need of a sudden high amount of energy to regenerate, will require a hierarchized process to maintain energy homeostasis. Understanding how this is done would help us understand how regeneration incompetent organisms could benefit from an adequate metabolism management.

## 2 Basic Systemic Regulation Of Metabolism

To maintain energy availability in all tissues, a vast network of regulatory checkpoints ensures homeostasis. Upon food ingestion, the main role of the digestive system is to obtain lipids, carbohydrates, proteins, minerals and vitamins. Although there is a significant interest in sugar and amino acid traffic, more recent work has re-started to look into lipids and uncovering novel roles in metabolism and signalling.^11–13^ First, the nutritional flow is regulated by intestinal cells after food ingestion. To obtain energy from the dietary components, complex dietary carbohydrates, protein and lipids, are broken down to ease their intestinal absorption, cellular transport and secretion into circulation. Nutritional flow continues with nutrient transport across barrier cells to reach cells of targeted tissues. In vertebrates, organs responsible for the regulation of metabolic homeostasis such as the liver, adipose tissue, pancreas and various glands, directly regulate the availability and use of nutrients and modulate anabolic or catabolic turnover. For the brain to exert control of homeostasis, integration of peripheral information is required. First, by direct innervation of these organs (e.g., direct innervation of the gut), the release of humoral factors into the bloodstream (e.g., leptin release by adipose tissue and insulin) and by the activity of dietary signal cues (e.g., glucose).^10^ Neural circuits sense and respond to the above-mentioned inputs, by commanding adjustments that will keep a balance between energy intake and energy expenditure. In the arcuate and paraventricular nuclei of the hypothalamus, neurons sitting in a highly vascularized area, directly sense nutrients and circulating hormones. In turn, proopiomelanocortin neurons (POMC) inhibit food intake and increase energy expenditure, or neuropeptide Y neurons (NPY) will direct food intake and inhibit energy expenditure (reviewed in Myers et al.,^10^). In addition, anabolic or catabolic turnover in dedicated storage organs (e.g., adipose tissue and liver) buffer the systemic availability of all essential cues required to allow optimal cellular functionality. These organs can also be regulated centrally, for example, sympathetic neurons stimulate the activity of adipocytes amplifying catabolic turnover to free fatty acids (FAs) from stores.^14^ While the storage of glucose (in the form of glycogen) is limited to small quantities, most energy resources are stored as fat in lipid droplets. Lastly, energy balance is influenced by other physiological systems such as the reproductive and stress axis (reviewed elsewhere^9^), and as proposed in this manuscript, by injury signals.

## 3 Metabolic Response And Adaptation In Injury

### 3.1 The injury message

The nervous, immune and circulatory systems convey and process information from the injury site. These systems are well studied independently, however, the interaction of multiple systems and interorgan communication is gaining attention. The central nervous system collects injury-related information via soluble factors,^15^ direct receptors that sense mechanical, thermal or chemical damage,^16^ or by directly regulating ion channels in nerve terminals. Specialised sensory neurons (nociceptors) detect stimuli that cause tissue damage and convey the information via the afferent neurons through the spinal cord, and then to the thalamus. The thalamus relays signals to the higher brain centres (the cerebral cortex), where the information is processed and results in an action (withdrawal from the stimulus), pain modulation and stress reaction (reviewed elsewhere^16^).

Injury-induced release of reactive oxygen species (ROS), triggers the first immune response recruiting inflammatory cells.^17^ Further immune system responds to damage-associated molecular patterns (DAMPs), released by damaged or dying cells, to ensure asepsis of the wound. Additionally, bacteria, pathogen-associated molecular patterns (PAMPs), metabolic by-products and cytokines also trigger an inflammatory response necessary to mobilise resources and restore homeostasis (reviewed elsewhere^18,19^). However, when an exacerbated response to a major injury is not controlled, secondary organ damage can occur even in locations remote from the injury, known as injury-associated systemic inflammatory response.^20^

Other less explored elements are locally released factors (e.g., metabolites) that enter the bloodstream. Together with DAMP signalling, such direct communication axes are able to convey injury signals into a systemic response and allow bidirectional communication between the site of injury and other body parts. Despite being well known that metabolites act as efficient signalling molecules, only little is known about the role of those locally released factors in wound repair and regeneration.^21^ However, it is likely that the interplay between the injured tissue, the immune system and the nervous system, is at least influenced by the various chemicals and metabolites produced locally.

### 3.2 Metabolic response to injury

Data from patients with severe trauma supports a significant metabolic re-adjustment. After injury, the organism responds with a switch to a catabolic state and hypermetabolism (elevated resting energy expenditure). In this situation, skeletal muscle is catabolised to support protein synthesis and, interestingly, the administration of nutrients is not sufficient to revert the catabolic state.^20,22,23^ In trauma patients with coma, hypermetabolism still occurs, suggesting that movement or awareness of the patient is not the cause. Moreover, metabolism after severe trauma is characterised by high cortisol, insulin resistance and hyperglycemia.^24,25^ Prolonged hyperglycemia and hypermetabolism has adverse effects for the healing process and could lead to further infections and even multiorgan failure. The type of injury is unlikely to be a determinant for a metabolic systemic response. For example, the resting metabolic rate is increased in patients with brain injury, burns and in skeletal trauma.^26^

### 3.3 Insulin

It is difficult to predict or compare the severity of the triggered hypermetabolism, as there is no comprehensive study analysing different types of injury and normalising for the severity of the trauma. A common denominator is however an acute insulin resistance, which seems to be unique to injury^25^ and likely to be the result of multiple factors. In patients with significant skin burns, the early phase post injury (hours) is characterised by high glucose levels and almost no response from the insulin signalling cascade. In a later phase (days and later) insulin levels rise, but blood glucose levels decrease only moderately, and heterotopic fat in various cell types including muscle and liver cells is observable. From the medical point of view, persistently high insulin reflects insulin resistance.^27^ In patients with brain trauma, metabolic dysfunction of brain cells adds to the damage, in the form of insulin resistance and decreased glucose uptake in undamaged brain areas,^28^ but also as a systemic effect.^29^ In a rat burn model, injury mimics the hyperglycemia and insulin resistance in patients. This response occurs as fast as 30-60 min after injury when trauma is combined with haemorrhage^30^ and helps to consolidate the response. In a rat model of surgical injury and haemorrhage, insulin signalling (IS) is affected in skeletal muscle as soon as 60 min post-surgery, reflected as a rapid decrease of AKT phosphorylation and insulin receptor function.^31^ Insulin resistance and hyperglycemia in turn, affect immune cells, favouring an inflammatory immune response.^32,33^ However, the interconnectivity and causation between insulin resistance, hyperglycemia and inflammation needs to be further investigated. In a tour de force study that mimics traumatic brain injury in Drosophila, Katzenberger et al., observe hyperglycemia in hemolymph and suggest that intestinal barrier dysfunction is a secondary trait triggered by injury.^34^

As dysregulation of IS has a direct impact on the patient’s recovery, there is a need to control alternations of insulin signalling resulting from injuries. Furthermore, the effects of IS on cellular decisions are evident. Glucose levels and IS are essential for stem cell division rates of Drosophila intestinal stem cells. The intestinal stem cell niche is formed by different cell types including visceral muscle cells. These cells produce the Drosophila insulin-like peptide 3 (DILP3) in response to intestinal damage and the availability of a calorie-rich diet. DILP3 is sufficient to promote stem cell division and its localised reduction minimises regenerative activity.^35^ Finally, diseases affecting metabolism (such as Diabetes) complicate the healing process including non-metabolic organs like brain, bones.^36^

### 3.4 Other humoral factors

Further evidence of a complex systemic response to injury is shown by Khallaf et al.^37^ The recovery of patients with traumatic brain injury (TBI), bone injury or concomitant TBI and bone injury was investigated. The group with combined injury showed accelerated bone healing, suggesting that humoral factors are involved. Indeed, an increase in growth hormone at 3 weeks after injury in the combined group is observed. In addition, an important systemic response to injury is the activation of the hypothalamic-pituitary-adrenal axis. Elevated corticosteroid levels were shown in patients of all groups. Moreover, within 24 h post injury in the group with the concomitant injury, a high concentration of the food intake and satiety regulator leptin was demonstrated. In support of a systemic effect, in a mouse model lacking leptin, the positive effect of the concomitant injury on bone healing is decreased.^38^

Others have shown that traumatic head injury increases the release of insulin-like growth factor 1 (IGF-1)^39^ and parathyroid hormone^40^ in circulation that could further explain the accelerated healing of bone. In addition, human and animal injury models have confirmed the consistent increase in cortisol in response to injury.^41^

Why these humoral factors are secreted is not fully understood, however, the consequences are various. For example, it has been proposed that insulin resistance and hyperglycemia are cortisol-induced. Although recent evidence shows that blocking the increase of cortisol in a rat injury model, does not prevent insulin resistance in the liver.^25^

### 3.5 Cellular mTOR signalling

The target of rapamycin (mTOR) regulates cell growth and metabolism through phosphorylation of kinases such as protein kinase C (PKC) and protein kinase B (AKT), and interacting with other subunits, it forms two functional complexes, mTORC1 and mTORC2. In response to nutrient availability, mTOR is activated in different injured tissues.^42,43^ The role of mTOR in skeletal muscle injury has been described as necessary. In the absence, or by blocking mTOR, muscle stem cells (satellite cells) fail to activate and proliferate.^43,44^ Rodgers et al. showed that mouse muscle injury on one side of the body triggers quiescent muscle stem cells on the contralateral side to enter an alert state, via mTOR.^45^ In addition, mTOR is required during tissue regeneration in planarian and zebrafish by regulating stem cell activation and blastema outgrowth.^46,47^ In salamanders, a subset of cells in the contralateral limb from an amputated animal, also express mTOR in response to limb regeneration.^48^

One possibility to explain a systemic response via mTOR is the fact that mTOR is merged with insulin signalling via the AKT-Ser473. In fact, mTORC1 is an effector of the insulin/PI3K pathway that promotes lipid synthesis, glycolysis and nucleotide synthesis.^49^ Moreover, activated AKT promotes the localization of the glucose transporter GLUT1 at the plasma membrane, ushering the cellular uptake of the sugar.^50^ Enhanced GLUT1-mediated glucose uptake is one prominent example of growth factor induced nutritional flow untethering cells from the regulation of whole-body glucose homeostasis.^51^

To fill local metabolic needs of healing tissues, a rather complex local and systemic regulatory network sets off to coordinate energy expenditure and availability. We are now starting to understand how a sustained healing, and highly proliferative process such as epimorphic regeneration, can maintain an active source of available fuel.

## 4 Bridge Keystones: Connecting Metabolic Regulators

### 4.1 Epimorphic regeneration

Epimorphic regeneration is characterised by anabolic metabolism; it requires building blocks and local metabolic adaptations to regrow the lost appendage. The highly proliferative cells of the forming blastema are associated with a metabolic switch to a more embryonic-like metabolic state. In lizard and *Xenopus* tadpole tail regeneration, blastema cells increase glycolysis and hexose monophosphate pathways.^52,53^ Similarly, in zebrafish metabolic reprogramming to high glucose uptake and lactate production (known as Warburg effect) is necessary for tail regeneration, and it is associated with the hexosamine biosynthetic pathway HBP and N-linked glycosylation.^54^

Proteomic analysis has shown a decrease of several enzymes of the citric acid cycle and oxidative phosphorylation in both regenerating *Xenopus* and axolotl.^55^ The regenerative environment is generally characterised by a local increase in reactive oxygen species (ROS) within hours post-amputation.^53,54^ ROS are produced as a byproduct of mitochondrial oxidative phosphorylation causing cell damage or by NADPH oxidases, particularly NOX2, enhancing immune defence and cell signalling functions.^56^ ROS are speculated to mediate glucose entry into glycolysis and the pentose phosphate pathway metabolism and to play a role in mammalian axonal regeneration through the NOX2-PI3K-p-AKT signalling pathway.^53,57^

These findings are supported by an increase in hypoxiainducible factor 1-alpha (HIF1α) activity induced by high ROS levels.^58^ HIF1α is a transcriptional master regulator that among many functions, induces the expression of VEGF, a key regulator of vasculogenesis and angiogenesis. In zebrafish and other vertebrates, vascularization of damaged heart tissue is one prerequisite to induce regeneration.^59^ This underlines how the vascular distribution, and thus oxygen and nutrient supply, during regeneration is intertwined with metabolism and stem cell function. Other less understood examples of ROS-induced tissue remodelling in response to wounding are reported in plants and flies. Plant leaves reconnect the wounded area shortly after tissue damage and an intense ROS production has been reported.^60,61^ Similarly, in the regenerating Drosophila gut, ROS induces vascular remodelling.^62^ Epimorphic regeneration also requires long-range factors like hormones that are locally upregulated. Notably, a local increase in the orexigen leptin and its receptor have been shown to enhance bone formation and healing of skin wounds.^63–65^ Although the role of circulating factors on leptin expression in the wound is not fully understood, there is evidence that cultured human fibroblasts secrete leptin in response to insulin.^66^ A further crosstalk with insulin signalling is observed intracellularly: leptin induces PI3K/AKT signalling, sharing the molecular cascade with the insulin receptor. At the injury site however, leptin is increased within early stages of *Xenopus* tail blastema and axolotl limb regeneration.^67,68^ It remains to be defined how the locally secreted leptin impacts neighbouring tissues or systemic leptin function.

Earlier studies have shown that salamander blastema cell proliferation and survival in vitro and in vivo depend on the combined presence of several hormones, like insulin or growth hormone (GH).^69,70^ Insulin, proinsulin and insulin-like growth factor (IGF) are upregulated in tail regeneration and are required for blastema cell proliferation^71^ and heart regeneration in zebrafish.^72^ Insulin/IGF-1 signalling activates downstream pathways that enhance cell survival, proliferation and differentiation through MAPK signalling, and PI3K-AKT signalling. This leads to lower injury-induced hyperglycemia, protection against elevated ROS and angiogenesis.^70^ Thus, insulin/IGF axis not only regulates energy balance, it also plays an important local role activating growth, osteogenesis, angiogenesis and stem cell survival.

Alpha melanocyte stimulating hormone (α-MSH) and its receptor melanocortin receptor 4 (MC4R) impact epimorphic regeneration both locally and systemically. α-MSH, a cleaved peptide from the precursor POMC, is released into circulation from neurons in the hypothalamus. Recently, it was shown in *Xenopus* limb regeneration, that α-MSH/MC4R were increased in blastema in a nerve dependent manner, and blocking the signalling of MC4R impairs regeneration.^73^ Using a crude hypothalamic injury, the authors observe a similar impact on regeneration, arguing for a local and systemic role for MC4R. Local α-MSH/MC4R signalling regulates ROS levels, ensures mitochondrial stress balancing the rate of glucose metabolism and promotes neurotrophic functions within blastema cells. Furthermore, MC4R knockout mice fail to regenerate the digit tip, while systemic α-MSH administration rescues regeneration in heterozygous mice. When applied in a proximal and regeneration incompetent digit amputation, α-MSH stimulates regeneration.^74^ Additional important factors linking metabolism and regeneration can be found in Table 1. Summarising, the local regenerative environment is characterised by changes in energy metabolism, in particular affecting glucose metabolism and IS. Evidence suggests that in addition, other systemic factors are crucial for epimorphic regeneration, likely induced by yet-unknown local key modifiers.

## 5 Lipids In Regeneration: The Unexplored Shore

### 5.1 Lipid circulation

In many animal species the bulk of endogenous lipids which enter circulation are produced by cells functionally analogous to intestinal cells, adipocytes or hepatocytes. All these cell types are able to secrete lipoprotein particles, systemic lipid carriers, or lipidated proteins destined to spread their lipid loads within the organism.^97^ The production of lipoprotein-lipid carriers is limited in type and location. Cells facilitate the uptake of lipoproteins or other lipid carriers initiating receptor mediated transport.^98,99^ These receptors belong to conserved protein families and their expression patterns are regulated by many factors including cell type, circadian rhythm and nutritional state.^100^ Stem cells are known to express different lipoprotein and albumin receptors (another major lipidated protein in circulation). It is likely that dividing cells in regenerating tissues require adequate lipids to facilitate their proliferation rates.^101^ Although most cell types are capable of producing lipid building blocks, such as FAs, to preserve time and energy lipids are absorbed from circulation. Animals absorb many dietary lipids; however, blood serum lipids represent a mixture of food lipids and endogenous lipids. The following sections are focused on bioactive lipids and lipid-like molecules of different origin.

### 5.2 Lipid and lipid-like cues in metabolism

Plants and some animal species rely on a steady nutritional flow to regenerate efficiently.^102,103^ However, plants are characterised by indeterminate post-embryonic development and the source for (re) generation of new organs are shoot or root stem cell niches—the so-called apical meristems. It is debated if these meristem cells are equivalent to stem cells in animals and if (re)growth of plants can be compared to animal regeneration.^104,105^ However, processes like the complete restoration of root-tips astonishingly mimic in many aspects the limb regeneration process in animals (reviewed in References 105,106). This highlights that despite mechanisms involved in regeneration between plants and animals that are likely to be different, the overall concepts and principles might be closely related and comparable. Remarkably, plants are able to absorb lipids from soil and are capable of long-range lipid transport, but it remains unclear to what extent and for which purpose plants possibly distribute lipids systematically.^107,108^

**Figure 1:**
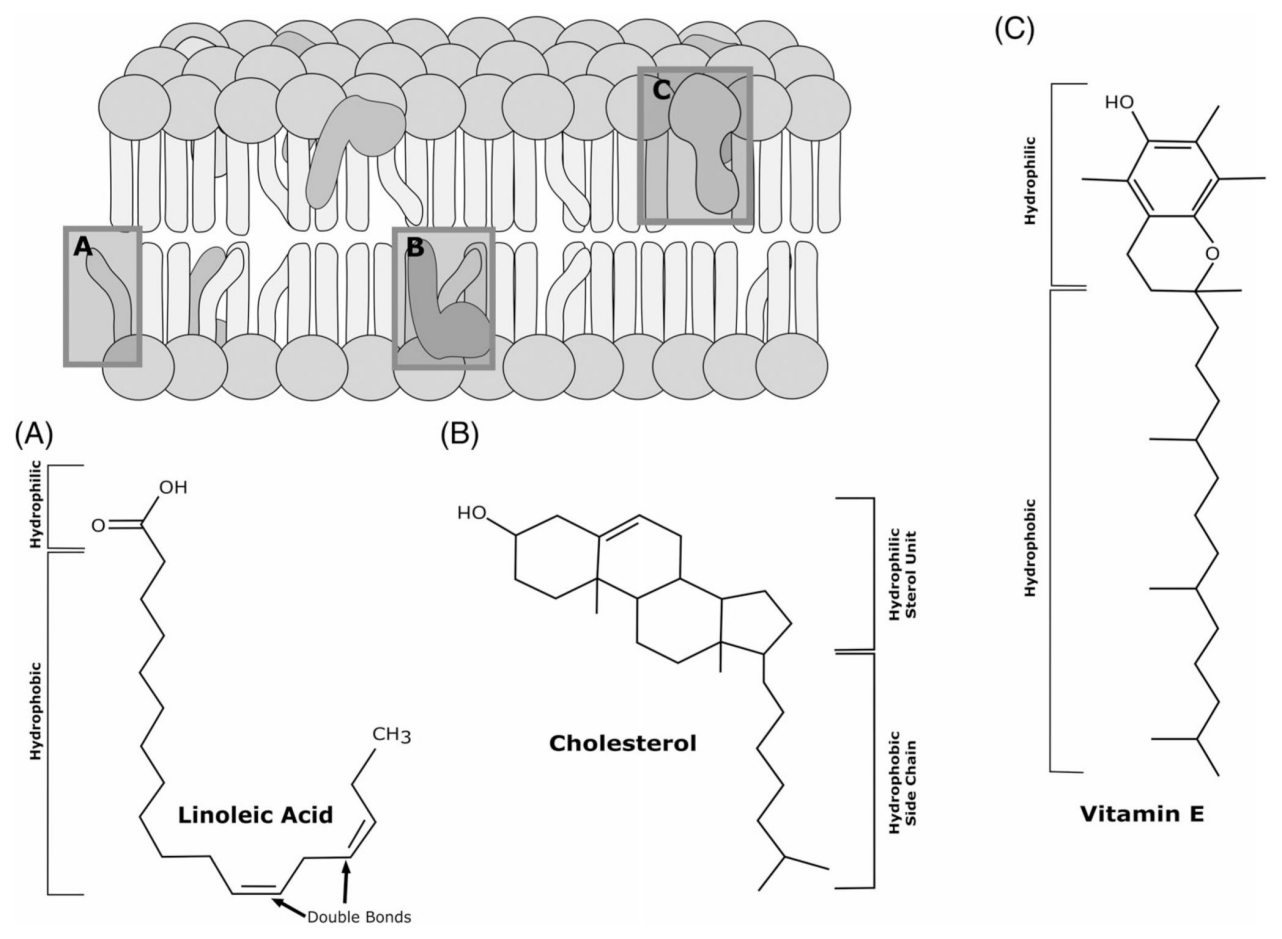
Fatty Acids, Sterols and Lipid-like Vitamins in their Role as Signalling Molecules and Membrane Components. Fatty acids (A) consist of a hydrophobic carbon chain with variable numbers of double bonds and a hydrophilic carboxyl (-COOH) group. The number of double bonds determines the degree of unsaturation. For example, (A) depicts the two times unsaturated fatty acid linoleic acid with a carbon chain length of 18. Fatty acids are usually not found in a free state but as phospholipids in membranes or in combination with glycerol as di- or triglycerides. In contrast, the lipid class of sterols (B) is characterised by a tetracyclic hydrophilic sterol unit and a hydrophobic carbon side chain. The main sterol in animals is cholesterol. Fatty acids and sterols are crucial structural components of all membranes and their proportion as well as degree of saturation results in altered cellular biophysical properties. Lipid-like vitamins, like vitamin E (C), can also influence membrane properties. Furthermore, lipids are important for energy storage, induce metabolic signalling and there is growing evidence on the role of lipids as signalling molecules in wound healing and regeneration.

In the past years, metabolic research retook the focus on the metabolic activity of dietary lipid-like molecules and lipids. Lipid-like vitamins, such as A, D and E, are essential to many animals and their biochemical properties allow such compounds to integrate into cellular membranes (Figure 1). As such, these vitamins are in direct contact with membrane lipids capable of interacting with the latter.^107^ For instance, the antioxidant vitamins E and A are believed to scavenge free radicals and thus, to protect the functional integrity of cellular membranes as well as to promote plasma membrane repair.^109–112^ In plants, vitamins regulate systemic metabolism and growth^6^ and interestingly, most media used in plant organ regeneration are supplemented with a range of these bioactive compounds. Although the vitamins A and E are enriched in many plant species, virtually nothing is known about their contribution in plant regeneration. Animals need to absorb vitamin A and E, and these two vitamins have very different biological roles. Vitamin A often gets converted into retinoic acid (RA). RA has many targets including genes involved in mediating canonical antioxidant responses, or nuclear retinoid receptors involved in the regulation of many systemic parameters including the glucose-lipid homeostasis.^113,114^ Moreover, it is known that RA is essential for patterning during salamander limb regeneration^115^ mediating the proximodistal specification in salamander limb regeneration.^116,117^ Furthermore, antagonising the retinoic acid receptors disrupts skeletal patterning and skeletogenesis during regeneration.^118^ In zebrafish, RA regulates proliferation and is required for blastema formation,^119^ as well as in heart regeneration, where injury-triggered RA controls cardiomyocyte proliferation.^120^ Not surprisingly, blocking the rate-limiting enzyme Raldh2, inhibits regeneration in zebrafish.^121^

Vitamin E is an important antioxidant and protects polyunsaturated fatty acids (PUFAs) in cellular membranes from oxidative destruction.^122^ PUFAs reduce ROS species efficiently, limiting the range of such signalling.^123^ Hence, vitamin E indirectly modulates metabolic rates since membrane integrated PUFAs shift the signalling capacity of many signalling pathways and the local lipid composition likely modulates vascularization. Studies suggest an inhibitory effect of vitamin E on liver regeneration in rats by altering the lipid peroxidation in the cytosol and plasma membranes.^124^ In contrast, vitamin D promotes regeneration of zebrafish fin^125^ and heart^126^ tissues as well as human skeletal muscle repair, and is necessary for skeletal tissue integration in salamander limb regeneration.^127^

Dietary lipids are capable of modulating the metabolism of consumers.^128^ Essential fatty acids (EFAs) are precursors for the generation of endogenous complex lipids acting as signalling molecules (e.g., PIPs), or can directly induce metabolic signals.^129^ In humans, EFAs need to be absorbed from food. Most studies focus on plant derived n-3 and n-6 omega FAs with a carbon chain length of 18-20 atoms, such as alpha-linolenic acid or linoleic acid (Figure 1). In regenerating organisms, EFAs contribute in several instances, including inhibition of oxidative stress,^130^ analgesic and anti-inflammatory properties.^131^ For instance, n-3 PUFAs are known to promote regeneration of several tissues such as muscles,^132^ liver cells,^133^ nervous system^134^ and endothelial cells.^135^ This list is by no means exhaustive, but demonstrates the broad target range of n-3 PUFAs. In contrast, n-6 PUFAs seem to inhibit the reparative potential of n-3 PUFAs.^136^ Nevertheless, some studies suggest that the chain length is another critical factor that modulates the bioactive potential of these lipids. For instance, in some plants very long FAs prevent the formation of regenerating tissue.^137^ Other examples are studies in rodents that report long EFAs promoting regeneration of various cell types^131^ and short saturated fatty acids (SSFAs) produced by intestinal microbes.^138^ Absorbed by host organisms, SSFAs are suggested to play many roles including regulating insulin signalling.^139^ The ratio between dietary SFAs/PUFAs changes the absorption rate of nutritional cues and metabolic rates, impacting on many parameters including lipid turnover (reviewed in References 140,141).

### 5.3 Endogenous signalling lipids linkup with the insulin signalling cascade

A less studied set of molecules in the context of repair are endogenous lipids regulating metabolic signalling cascades. However, what we currently know poses them as an important player. Starving organisms lower their metabolic rates and many enter dormancy-like states to endure long periods of food deprivation. Nevertheless, some species maintain their regenerative capacity despite none or insufficient food consumption.^142,143^ It appears that fasting or fasting mimicking diets can sensitise metabolic pathways^144^ and promote regeneration.^145^ For instance, in response to food deprivation, adipose tissues in mice produce and secrete lipid signalling cues named PAHSAs (Palmitic acid esters of hydroxystearic acid). Some PAHSA-lipid species promote insulin release into the blood system.^146^ However, dietary supplementation of these lipids had no effect on IS.^147^ Fasting also changes the activity of neuronal subsets wired with organs responsible for the redistribution of nutritional molecules.^148^ For example, free FAs that induce signalling in hypothalamic neurons or glia.^149^ Starving cells dismantle endogenous complex lipids to fill energy demands and produce free FAs fit for mitochondrial reduction. Free FAs activate neurons wired with hepatocytes and in response, the liver cells start to release hepatic glucose into the blood system, essential to maintain basic blood-glucose levels.^150^

Another factor important to facilitate IS are sterol levels, as the activity of the mammalian insulin receptor depends on membrane sterols^151^ (Figure 1). Extending the concept to sterol auxotrophs, dietary sterol yields could prove essential for IS and cell proliferation rates.^152^ Sterols are precursor molecules for the synthesis of many steroids and steroid hormones. Moreover, in plants, Drosophila and murine models it was shown that the activity of insulin producing cells is dependent on steroid hormones.^153–155^ In addition, steroids regulate the expression of insulin binding proteins that deactivate the hormone.^156,157^ In sterol auxotrophs, the quality of steroid hormones is defined by the identity of dietary sterols,^158,159^ however, steroids production is sex specific. Indeed, in humans, steroids are responsible to modulate sex-specific insulin sensitivity and regeneration potential in muscle.^160^

Taken together, the role of endogenous lipid cues potentially reaches far beyond the conservation and distribution of energy. We propose that endogenous lipid cues produced in response to metabolic changes are important factors regulating IS during regeneration. Therefore, we single out such lipid cues as rewarding targets to our understanding of systemic metabolism in the context of regeneration.

## 6 Outlook

### 6.1 Nutrition: Food and feeding behaviour

**Figure 2:**
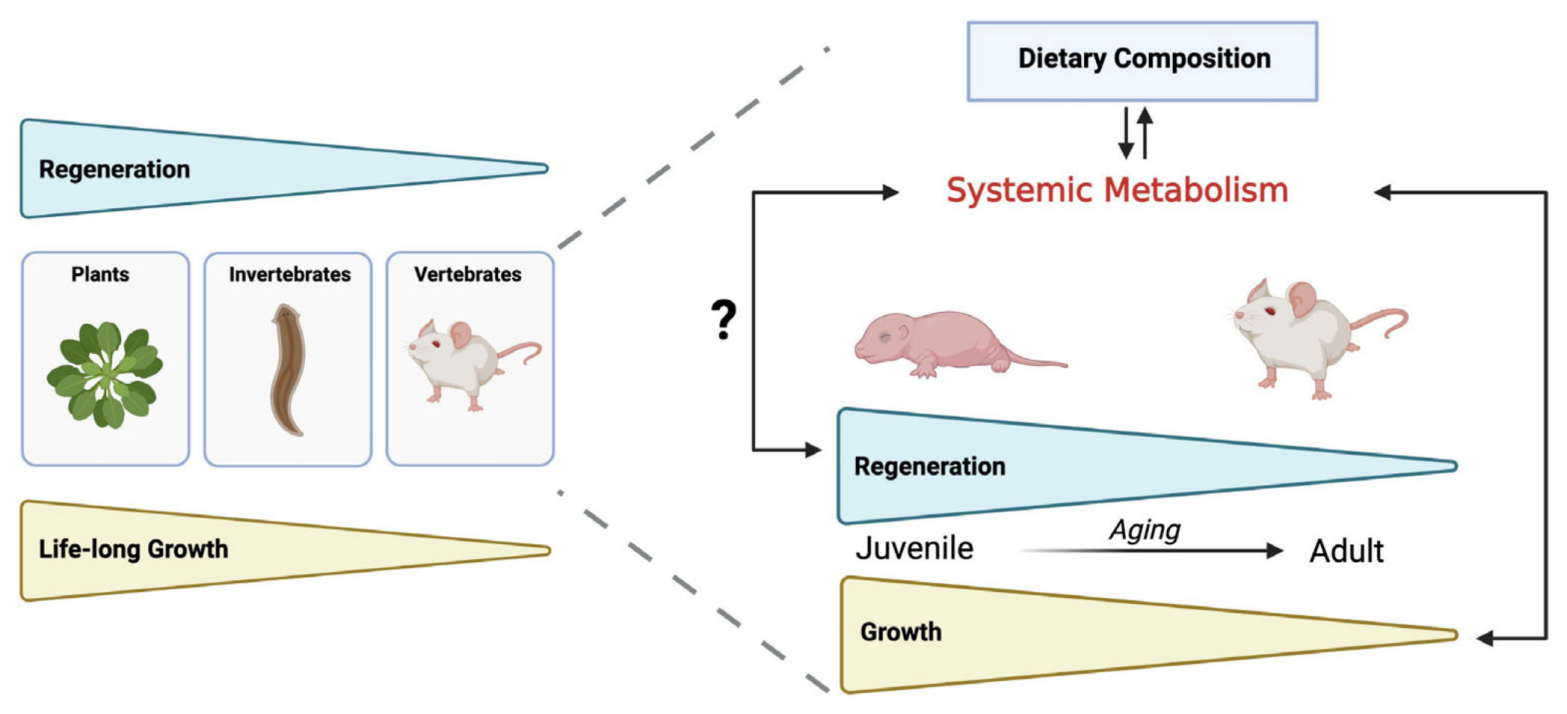
Connection of Regeneration, Growth, Aging and Systemic Metabolism. The regenerative potential varies greatly between species but tends to be lowest in vertebrates compared to plants and invertebrate species. The regenerative potential tends to decrease during aging and it has been shown that the nutritional quality and flow are age dependent. However, how the systemic metabolism impacts the regenerative abilities remains widely unexplored to date. Nevertheless, systemic metabolism and nutritional changes have the potential to be one key variable to promote regenerative tissue repair. Created with BioRender.com.

Absorbed dietary lipids can induce metabolic signalling, are structural units integrated into membranes which results in altered cellular bio-physical properties (Figure 1) and provide energy. Thus, the regenerative potential of tissues could profit from the quality and quantity of ingested lipids. In addition, dietary vitamins, sugars and proteins represent additional nutritional factors, especially since excess sugars are converted into lipids. However, the nutritional quality and flow are age dependent, as food preference, feeding frequency and energy management change with age.^161,162^ Nutritional changes potentially modulate the regeneration capability, as an example, salamanders, after a severe period of starvation can still form a blastema but fail to re-grow the limb^163^ (Figure 2).

The extraction of nutrients from ingested food is also dependent on the intestinal food transition time and on the activity of intestinal microbes. Many examples are known about intestinal microbes producing metabolites capable of modulating the metabolic rate of the host. Especially the microbial fermentation of dietary fibres results in the production of short fatty acids that regulate physiological values such as blood pressure,^164^ nutritional absorption,^165^ modulate behavioural variables^166^ and to change the host metabolic rates.^167^ All these parameters are important for adjusting screws directly or indirectly controlling the regenerative potential.

### 6.2 Regeneration: Systemic metabolic cues

We have provided relevant literature of multiple factors that circulate and reach areas distant to the injury site. This systemic response modulates cellular decisions with impactful consequences. While currently we lack the direct connections from the local response to the systemic response, it is possible to speculate that such signals are designed to craft a network of inter-organ communication and orchestrate a systemic adaptation.

Such messenger molecules can range from simple turnover metabolites, such as lactate, to specialised proteins produced specifically in response to tissue repair. Another possibility are factors that participate in homeostasis and then upon injury, acquire a transient role. One example of the latter category is leptin. It is certainly interesting to consider if leptin released by regenerating tissues hijacks the canonical leptin signalling and increases circulating lipid levels, while promoting healing locally.

### 6.3 Regeneration: Invasive microbes and microbiota

Tissue injury is often associated with microbial contamination and thus, an acceleration of the host immune-system activity is inevitable. The intensity of the immune response depends on many factors including the type of invading microbes, microbial products, infected area and wound closure.^56^ Interestingly, some elements produced by activated immune cells modulate systemic metabolic rates^168,169^ likely to redirect nutritional flows. Hence, the immune response associated with extensive tissue damage could represent a critical metabolic switch, instructive in the decision to initiate tissue repair by regeneration or perhaps scar formation.

The immune and metabolic systems greatly benefit from the symbiosis of microbes in the homeostasis of organisms, and how they could have a remote effect on regeneration is a fascinating new aspect that is gaining deserved attention.^170^ In particular, microbiota could influence systemic inflammation and indirectly, insulin resistance in humans.^171^

### 6.4 Regeneration: Biomedical implications

The modulation of the nutritional flow represents a plausible field to potentiate regenerative tissue repair. Therapeutic interventions at different levels focusing on bioactive dietary lipids and acute glucose homeostasis are imperative. Another therapeutic level to foster regeneration are strategies aiming at the physiology of affected organisms. More invasive methods are ideas involving targeted stimulation of organs key for metabolic controls (e.g., liver/adipose tissue) or microbial treatments aiming to optimise nutritional absorption, potentiate the immune response or provide microbial metabolites catalyzing metabolic signalling. In addition, ongoing research on insulin signalling and the metabolic shift of immune and stem cells close to tissue damage may result in therapeutic strategies including patients suffering from metabolic diseases like Diabetes.

### 6.5 Final remarks

In this review we have investigated the possibility that systemic metabolism is one key variable to promote regenerative tissue repair. We propose that regenerating cells do not simply rely on local signalling cues and sufficient nutritional provision, but actively influence other tissues responsible for the mobilisation of molecular building blocks, such as lipids. Future coordinated studies into the topic across different species will reveal conserved molecular mechanisms key to formulate biomedical strategies aiming to improve wound healing.

## Figures and Tables

**Table 1:** Additional signals/processes linked to energy metabolism in repair and regeneration

Signal/process	Effect	Species
Initiation of regenerative activity immediate after injury		
mTOR signalling	Neurite regrowth.	Rat^75^
	Satellite cell activation.	Mouse^44^
Cell division rate/Growth		
ROS	Apoptosis and JNK signalling promoting epidermal proliferation.	Zebrafish^76^
Insulin	Regulation of division rates in intestinal and germline stem cells.	Drosophila^77,78^
Leptin	Enhances proliferation, differentiation and migration of epidermal keratinocytes in wound healing.	Human^63^
MC4R/α-MSH	Induces proliferation during the early regeneration phase.	*Xenopus*, Mouse^74^
PPARα and ß/δ	Regulates proliferation via induction of AKT signalling.	Zebrafish^79^ Mouse/Rat^80^
Energy homeostasis adaptations		
↓Peroxisome proliferator-activated receptor gamma (PPARy) and sphingosine-1-phosphate (S1P)	Lipidome adaptations after resection/cell culture injury in Schwann cells.	Mouse^81^
GLUT1 transporter	Switches to glucose metabolism after *cryo-injury* of the heart.	Mouse^82^
Yin Yang1 (YY1)	Facilitates a metabolic reprogramming of satellite cells toward glycolysis.	Mouse^83^
ROS	Reduces oxidative metabolism due to lower mitochondrial activity in liver regeneration.	Mouse^84^
↑Regenerating Islet- Derived (Reg) proteins	Enhances liver and neuronal regeneration likely through insulin signalling downstream of the InR (AKT, pMAPK).	Conserved^85^
MC4R/α-MSH	Regulates energy homeostasis/body weight and reduces mitochondrial stress during the early regeneration phase.	*Xenopus* ^73,74^
PPARα	Regulates lipid metabolism maintaining on-going FA oxidation.	Mouse^86^
PPARß	Regulates AKT/E2f mediated FA synthesis and glycolysis.	Mouse^87^
Cell differentiation/patterning		
Leptin	Promotes osteogenesis. Increased leptin-induced osteogenesis via brain injury.	Human,^88^ Mouse^89^
TGF-ß	Promotes blastema formation via induction of epithelial to mesenchymal transition (EMT).	Zebrafish^54^
Auxin	Polarisation signal that induces PIN1 auxin transporter-marked channel formation during (vascular) cambium regeneration and vasculature formation.	Arabidopsis^90^
Vegfα	Promotes revascularization of the damaged heart, in its absence, proliferation is inhibited.	Zebrafish^59^
Resources allocation/storage sites		
Insulin	Trophic effect of insulin in hepatic regeneration.	Rat^91^
Adipose tissue	Secretion of adipokines may influence insulin resistance in critical illness.	Human^92^
Lipid/energy storage sites	Unrestricted food can compensate for growth limitations of regenerating juveniles.	Gecko^93^
Obesity	Ectopic adipocyte accumulation in bone marrow reduces haematopoietic regeneration.	Mouse^94^
Obesity	Decreases regenerative potential of muscle cells.	Human^95^
Hyperglycemia	Decreased fin regeneration.	Zebrafish^96^

## Data Availability

Data sharing is not applicable to this article as no new data were created or analyzed in this study.
